# Comparative Efficacy of Jaungo, A Traditional Herbal Ointment, and the Water-in-Oil Type Non-Steroidal Moisturizer for Radiation-Induced Dermatitis in Patients With Breast Cancer: A Study Protocol for a Prospective, Randomized, Single-Blinded, Pilot Study

**DOI:** 10.3389/fphar.2021.751812

**Published:** 2021-09-21

**Authors:** Eun Hye Kim, Jee-Hyun Yoon, Su Bin Park, Jee Young Lee, Weon Kuu Chung, Seong Woo Yoon

**Affiliations:** ^1^ Department of Korean Internal Medicine, Korean Medicine Cancer Center, Kyung Hee University Hospital at Gangdong, Seoul, South Korea; ^2^ Jaseng Spine and Joint Research Institute, Jaseng Medical Foundation, Seoul, South Korea; ^3^ Department of Radiation Oncology, Kyung Hee University at Gangdong, Seoul, South Korea

**Keywords:** Jaungo, Shiunko, radiation-induced dermatitis, traditional medicine, breast cancer

## Abstract

**Background:** Radiation-induced dermatitis (RID) is a common complication of radiation therapy (RT). Although it has a high prevalence and can even trigger the premature end of conventional cancer therapies, there is no standard management. This study aims to evaluate whether topical use of Jaungo (Shiunko), a traditional herbal ointment mainly composed of *Lithospermi radix* and *Angelica sinensis*, could reduce RID compared to the water-in-oil type non-steroidal moisturizer in patients with breast cancer.

**Methods:** This is a prospective, single-blinded, randomized controlled pilot trial that investigates the effect of topical application of Jaungo for the prevention of RID in postoperative breast cancer patients scheduled for RT, in comparison with the non-steroidal moisturizer, with a random distribution of 50 patients across the two groups. RT will be administered for 5–7 weeks with a biological equivalent dose (BED_10_) of 60 Gy or more, and the interventions will be applied 3 times a day during RT duration. Participants will be assessed a total of nine times, including eight visits during the period of RT and one visit at a 2-week follow-up period after the end of treatment. The incidence and severity of RID, quality of life, skin reaction symptoms, and maximum pain related to RID will be measured. The incidence rate of grade 2 or higher RID using the Radiation Therapy Oncology Group (RTOG) in the two groups will be statistically compared as the primary outcome. The types and frequencies of adverse events will be also collected and evaluated. All assessments will be performed by independent radiology oncologists.

**Discussion:** This trial is currently ongoing and is recruiting. This study will determine the preventive efficacy of Jaungo in RID with postoperative breast cancer patients and provide evidence in traditional Korean medicine clinical practice.

## Introduction

Radiation-induced dermatitis (RID) is one of the most common complications of radiation therapy (RT) in patients with breast cancer (Bolderston et al., 2006), and it presents with a variety of symptoms such as pruritus, erythema, edema, desquamation, necrosis, ulceration, and hemorrhage. It has been reported that nearly 90% of patients undergoing RT for breast cancer develop erythema-symptomatic RID (Geara et al., 2018). RID can induce significant discomfort, including fibrosis and skin pigmentation, negative effects on quality of life, and may even delay or prematurely end conventional cancer therapies such as RT or chemotherapy (Iacovelli et al., 2020).

Although several interventions for preventing or reducing RID, including systemic oral medications, skin care practices, steroidal topical therapies, non-steroidal therapies, and dressings have been evaluated, there is no standard treatment that has clearly demonstrated its effectiveness (Chan et al., 2014). However, in the clinical setting, skincare practices such as gentle washing with soap, or applying a moisturizing cream are generally recommended for patients with RID, even though there is insufficient evidence supporting their efficacy (Wong et al., 2013; Chan et al., 2014).

A water-in-oil type non-steroidal moisturizer containing hyaluronic acid as the main component has shown potential as effective in preventing and minimizing RID in patients with breast cancer compared to an emollient base cream regarding the maximum severity of skin toxicity, burning, and desquamation (Primavera et al., 2006). It has been reported to act as a barrier to the irradiated area, as well as having wound regeneration and anti-inflammatory effects (Leonardi et al., 2008).

Jaungo (Shiunko in Chinese), a traditional herbal ointment mainly composed of *Lithospermi radix* and *Angelica sinensis*, has been commonly used to treat wounded skin caused by cuts, abrasion, frost, or burn (Huang et al., 2004). *Lithospermi radix* contains shikonin, acetylshikonin, and dimethylacrylshikonin, and *Angelica sinensis* contains ferulic acid and decursin as the major active components. These components have therapeutic effects on granulation tissue formation, speeding wound healing, reepithelization, and angiogenesis in addition to antibacterial and anti-inflammatory effects (Lu et al., 2008; Andújar et al., 2013; Ghaisas et al., 2014). Recent case series have reported that Jaungo showed favorable effects on radiation-induced scalp dermatitis in patients with brain tumors (Hayashi and Sato, 2011), and a previous clinical study showed that Jaungo reduced the incidence of RID in comparison with gentle washing with neutral pH soap with no safety issues in patients with breast cancer (Kong et al., 2016). However, the comparative clinical trial of Jaungo and the water-in-oil type non-steroidal moisturizer was not conducted.

Given the high prevalence and limited therapeutic options of RID, comparative studies between the topical agents which are recommended clinically to prevent RID in patients with breast cancer are needed. This is the first randomized controlled trial which aims to evaluate the preventive efficacy of adjuvant topical application of Jaungo for RID in breast cancer patients compared to the water-in-oil type non-steroidal moisturizer.

## Methods and Analysis

### Study Design

**FIGURE 1:**
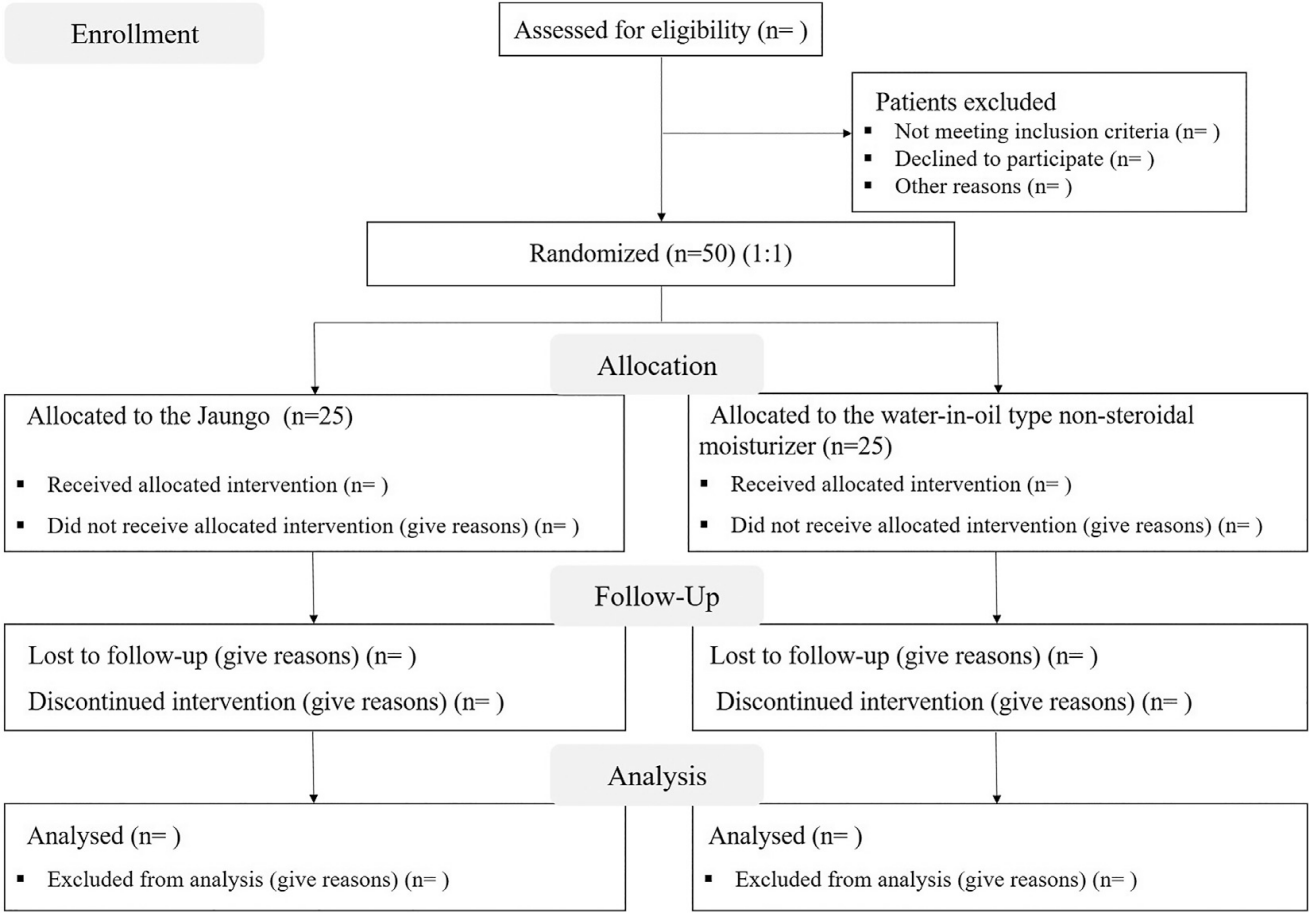
SPIRIT flow diagram of the study protocol.

This is a single-center, prospective, single-blinded, pilot randomized controlled trial protocol, which aims to investigate the comparative efficacy of Jaungo (Hanpoong Pharm & Foods Corporation, Seoul, South Korea) and the water-in-oil type non-steroidal moisturizer (X-derm®, Pharmbio Korea Inc., Seoul, South Korea) at preventing RID in patients with breast cancer who underwent breast-conserving surgery. This protocol is based on the Standard Protocol Items: Recommendations for Interventional Trials (SPIRIT) (Chan et al., 2013). The SPIRIT flow diagram of the study is shown in Figure 1.

### Ethics and Registration

The study was approved by the Institutional Research Board of Kyung Hee University Hospital at Gangdong, Seoul, South Korea (IRB number KHNMCOH-2020-12-001-002). This protocol has been registered on the Clinical Research Information Service (CRIS registration number KCT0005971), and the study will be conducted at the Kyung Hee University Hospital at Gangdong. We will fully inform the patients about the potential risks of this trial. Participants need to sign a written informed consent form to show that they understand the study program and are willing to enroll in the trial. They are free to choose whether to continue the trial at any time during the study.

### Sample Size

In this pilot study, the sample size was estimated according to the grade (Gr) of the Radiation Therapy Oncology Group (RTOG) (Cox et al., 1995) and National Cancer Institute (NCI) (Trotti et al., 2000) for the RID reported in the relevant literature. Kong et al. (Kong et al., 2016) investigated the incidence rate of RTOG Gr ≥ 2 in breast cancer patients with RID using Jaungo, which is the primary outcome in this study, at 46.7%. For non-steroidal moisturizer, there was no primary report using RTOG grade, but the incidence rate of NCI Gr ≥ 2 of RID in breast cancer patients was 9.0% (Leonardi et al., 2008). Clinically, the RTOG and NCI are common assessment grading tools, and each value of the incidence rate is compatible. Therefore, we estimated the incidence rates of the Jaungo and the moisturizer groups to be 0.467 and 0.09, respectively. The formula for calculating the sample size is as follows:

$$n_{c}=\;\frac{\left(1+\lambda \right)\sigma^{2}{\left(Z_{\alpha /2}+Z_{\beta }\right)}^{2}}{\lambda d^{2}}$$

At the 5% significance level, considering an estimated withdrawal rate of 15%, a total of 50 participants, 25 in each group will be required to achieve 80% power.

### Patients

#### Inclusion Criteria

1) Adult participants who are at least 19 years old or older; 2) subjects who were diagnosed with invasive breast cancer and recommended surgery for breast cancer; 3) subjects who received breast-conserving surgery; 4) subjects who were recommended to receive adjuvant RT after surgery; 5) Eastern Cooperative Oncology Group (ECOG) performance status (Oken et al., 1982) 1 or below; and 6) understanding of the study and willingness to provide signed consent to participate.

#### Exclusion Criteria

1) Cancer invasion to skin (T4 in eighth American Joint Committee on Cancer (AJCC) (Amin et al., 2017) TNM staging); 2) unhealed scar on the breast; 3) metastatic stage; 4) subjects who have a history of RT in the thoracic area for any reason; 5) soft tissue disease; 6) uncontrolled diabetes mellitus with an HbA1c value of 6.5% or greater; 7) subjects who are now or planned to be pregnant or breastfeeding during the study schedule; 8) subjects who are allergic to the medication; 9) Low compliance with any reason, including communication failure; and 10) abuse of alcohol or medication.

#### Prohibited Concomitant Medications

1) Intravenous antibiotics; 2) steroidal medication; 3) vasoprotective medication; 4) other treatments related to RID, except for the intervention, such as traditional Korean medicine (acupuncture, moxibustion, and herbal medicines), self-treatment (parse, vitamins, health-functioning foods, general medicines, and home remedies); 5) no other topical ointments or creams should be applied to the irradiated lesion, except for regular moisturizing lotion.

#### Drop-Out Criteria

Any patient who meets one of the following conditions will be dropped out from the trial. 1) The subjects or their families withdraw consent to participate in the trial; 2) a violation of the inclusion/exclusion criteria is found during the trial; 3) a significant adverse event appears in the subject and/or it is difficult to continue the trial due to an adverse reaction; 4) any violation of the study protocol; 5) the compliance level of the intervention (Jaungo or moisturizer) is less than 70%; 6) subjects who take medications, etc. that may affect the results of this study without confirmation from the attending physician; 7) any cases that the attending physician judge as inappropriate to the trial; 8) subjects who pregnant during the trial period; 9) subjects who fail to follow up during the trial period; 10) subjects who died during the trial period.

The methods used to contact the subject must be recorded in the supporting documents in the case of subjects who fail to follow up to prove that reasonable efforts were made to contact them. The patients who dropped-out due to significant adverse events could be provided with appropriate treatment for side effects. In these cases, the attending physician shall continuously evaluate the subjects until the adverse reactions are resolved or determined to be permanent.

### Randomization and Blinding

Patients who have agreed to the trial will be screened on the day of screening (visit 0) whether they meet the inclusion/exclusion criteria. The enrolled participants will be randomly allocated to two parallel groups with a 1:1 ratio at the date of visit 1; either the Jaungo group or the moisturizer group. The random number will be generated by an independent statistician using a 2 × 2 randomized permuted block design. Each piece of paper with the allocation group information according to the random number will be sequentially inserted into an opaque envelope and sealed. After the screening, the enrolled participants will open the envelopes in order and will thus be assigned to either the Jaungo group or the moisturizer group.

Throughout the study process, the clinical research coordinator is responsible for recruiting participants, screening, and assigning random assignments. This trial is a single-blinded study because each intervention can be distinguished differently from the participants. The statisticians and envelope processors, group allocators, management pharmacists, and outcome assessors for the clinical trial are all independent. Additionally, the principal investigator and sub-investigator will be blinded to the assigned group.

### Treatment Protocol

#### Radiation Therapy

All subjects will receive RT, regardless of the type of intervention. Patients will undergo 3-dimensional conformal RT (3D-CRT) or intensity-modulated RT (IMRT) for a maximum period of 7 weeks after breast cancer surgery according to the National and International Guidelines. The tumor bed will be treated with a biological equivalent dose (BED_10_) of 60 Gy or more, with a schedule of five fractions weekly for 5–7 weeks. In some cases, regional lymph nodal irradiation will be delivered with risk factors for regional recurrence, such as invasion of lymphovascular or axillary lymph nodes.

#### Intervention

The research will be conducted for a total of 7–9 weeks with nine individual assessments, consisting of topically administrating the Jaungo (Hanpoong Pharm & Foods Corporation, Seoul, South Korea) or the moisturizer (X-derm^®^, Pharmbio Korea Corporation, Seoul, South Korea) for the period of RT, 5–7 weeks, through eight visits after screening and a follow-up 2 weeks after the end of the application. The participants assigned to the Jaungo group will apply Jaungo on the entire irradiated skin region three times a day from the first day of RT, with a 1 cm distance from the skin marking line. The moisturizer group will apply the moisturizer in the same process.

#### Contraindications of Intervention

1) The intervention medications should not be used in patients with a history of hypersensitivity to the drugs, severe or extensive trauma, high fever in the suppuration area, and severe eczema or maceration in the radiated lesion; 2) in the case of skin irritation, local rash, or itching during the use of intervention medications, the application should be stopped; 3) the intervention medications should only be used as topical agents; 4) the intervention medications should be stored in a cool place away from direct sunlight.

### Outcome Measurement

The participants will be assessed at every visit during RT and 2 weeks after the end of RT. The assessment will be conducted by radiation oncologists who do not know which group the participant belongs to, and will be single-blinded. The date of the occurrence and severity of RID will be evaluated at every visit from the start to end of RT and 2 weeks after, based on the RTOG grade. An incidence rate of Gr ≥ 2 RID will be recorded as the primary outcome of this study. At the start and end of RT and 2 weeks later, the quality of life (QOL), skin reaction symptoms, and maximum pain related to RID will be measured based on the Catterall skin scoring profile (CSSP) (Catterall et al., 1971), the Korean version of Skindex-29 (Ahn et al., 2004), and numeric rating scale (NRS) (Haefeli and Elfering, 2006). The maximum grade of RID, time to onset of RID, the value of CSSP, the Korean version of Skindex-29, and NRS, and the types and frequencies of adverse events will be compared as secondary outcomes between the two groups.

#### Demographic Characteristics

Sex, age, ECOG performance status, and history of the disease are included in the demographic characteristics.

#### RTOG Grade

The RTOG toxicity scale was developed by the Radiation Therapy Oncology Group in the United States, which measures the grade of skin tissue damage caused by RT (Cox et al., 1995) and is classified into 0–4 grades according to the symptoms of dermatitis: Gr 0, no change over baseline; Gr 1, follicular, faint or dull erythema, epilation, dry desquamation, decreased sweating; Gr 2, tender or bright erythema, patchy moist desquamation, moderate edema; Gr 3, confluent, moist desquamation other than skin folds, pitting edema; Gr 4, ulceration hemorrhage, necrosis.

#### Catterall Skin Scoring Profile

The CSSP was developed by Fowler to allow the evaluation of skin reaction in mice, and was later modified by Catterall in 1965 to apply a numeric scale-type assessment tool (Catterall et al., 1971). It is classified as 1–10 points, depending on the symptoms and severity of dermatitis: 1, no visible reaction; 2, light erythema; 3, moderate erythema; 4, severe erythema; 5, dry desquamation <50% of irradiated are; 6, dry desquamation >50% of irradiated area; 7, blistering present; 8, moist desquamation <50% of the irradiated area; 9, moist desquamation >50% of the irradiated area; 10, ulceration present.

#### Skindex-29

Skindex-29 is a revision of the existing 61 questions of Skindex to 29 questions for patients with skin diseases. Skindex-29 is a QOL assessment tool designed to evaluate the QOL of a patient on three scales of symptoms, function, and emotion, while maintaining excellent reproducibility, reliability, recruitment validity, and content validity of Skindex. In this study, we will use the Korean version of Skindex-29 published by Ahn et al. (2004), which is a 5-level Likert scale from 1 (never) to 5 (always) and has been validated.

#### Numeric Rating Scale

NRS is a pain evaluation tool that uses the visual pain score (Haefeli and Elfering, 2006). NRS 0 is defined as the state without discomfort, and NRS 10 is the maximum degree of discomfort that the patient can think of.

#### Adverse Events

Clinical adverse events will be evaluated based on the Common Terminology Criteria for Adverse Events (CTCAE), version 5.0, developed by the NCI (Freites-Martinez et al., 2021). The research manager will check for adverse events during the period of intervention medication use at each visit. Information on adverse events is provided to participants reporting to the research manager, and the manager checks the occurrence of adverse events through a questionnaire at each visit. The notified events will be recorded in the “Adverse Reaction Record Table”. Adverse events will also be reported in detail on the date of onset and disappearance, the severity and outcome of events, the treatment taken in relation to the events and the causal relationship with the intervention, and the name of the suspected drug other than the intervention.

### Data Collection and Management

**FIGURE 2:**
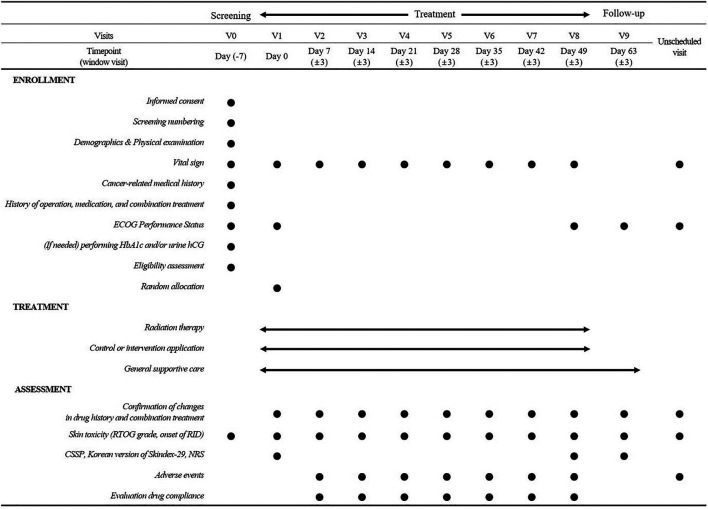
Study procedures and outcome measurements.

The participants will visit the outpatient clinic every week with a 3-day visit window during RT and 2 weeks after the end of RT. Figure 2 shows the study procedure and outcome measurements.

One or two assistants will collect and record the entire data in electronic case report forms. Personal information will be stored in an independent storeroom to protect confidentiality. The contract research organization (CRO) will monitor the data, protocol, and safety of this study and check the source documents every month. Access to the online database will be restricted to radiology oncologist researchers in this study team, as well as non-related outside people.

### Statistical Analysis

The collected data will be statistically assessed using SPSS Version 25.0 (Chicago, IL), based on the intention-to-treat (ITT) set, except for the following subjects: 1) subjects who violate the inclusion/exclusion criteria; 2) subjects who have never used the intervention medication; and 3) subjects who have not provided any data after screening. The demographic characteristics of all participants will be reported as the mean and 95% confidence interval (95% CI) for continuous variables or frequencies and percentages for categorical variables. The incidence rate of RTOG Gr ≥ 2 RID, the primary outcome of this study, will be evaluated the difference between two groups using the Chi-squared test. Among the secondary outcomes, the maximum grade of RID based on RTOG will be analyzed by Chi-squared test, the time to RID occurrence by Kaplan-Meier analysis, and the CSSP, Skindex-29, and the maximum NRS for RID-related pain by independent sample t-test or Mann-Whitney U test, depending on the normal distribution, to compare the differences between the two groups. Adverse events will be presented as descriptive statistics by dividing according to the severity of each intervention and adverse symptoms. The types and frequencies of adverse events determined to be causative of the intervention will be separately analyzed and reported. Differences will be considered statistically significant when *p* < 0.05. Adverse reactions will be presented as descriptive statistics, divided according to the severity of each group and each adverse event. The symptoms and frequencies of adverse reactions related to the intervention medication will be analyzed separately.

## Discussion

This is a prospective, single-blinded, pilot randomized-controlled trial to compare the preventive efficacy of Jaungo ointment and the water-in-oil type non-steroidal moisturizer for RID in patients with breast cancer undergoing RT after surgery. The number of participants was conducted for a total of 50 subjects, 25 in each group, based on prior studies. One of the two topical agents (Jaungo or moisturizer) will be randomly applied to the irradiated lesion from the start to the end of RT. In this protocol, the incidence and severity of RID and RT-related skin reactions, QOL, and pain based on RTOG, CSSP, the Korean version of Skindex-29, and NRS will be measured. The primary outcome will be the incidence rate of RID of Gr ≥ 2 based on the RTOG. All assessments will be conducted by independent radiation oncologists, blinded to the group.

RT is a widely applied conventional therapy for patients with cancer. Indeed, at least 50% of patients with any type of cancer receive RT during their treatment, and this prevalence is higher in patients with breast cancer up to 70% (Baskar et al., 2012). Almost 95% of patients receiving RT experience RID during or after the course of therapy, and 25% develop severe skin reactions (Iacovelli et al., 2020). The pathogenesis of RID is associated with radiation-induced vascular damage, followed by leukocyte infiltration and destruction of the skin barrier, resulting in inflammation and vasculitis (Weintraub et al., 2010). Patients may experience a variety of symptoms, such as pruritus, erythema, edema, desquamation, necrosis, ulceration, and hemorrhage. Furthermore, one out of three patients with breast cancer develop chronic RID, which can appear up to 10 years after the completion of RT. As such, RID is one of the most widespread side effects of conventional cancer therapies and has major consequences on QOL and adherence to RT course and dosage of irradiation, thereby even discontinuing RT and adversely affecting clinical outcomes (Leventhal and Young, 2017).

The risk of developing RID in breast cancer is dependent on various factors. Therapy-related factors include radiation dose, duration of irradiation, volume of the breast, and combination with other conventional therapies. The incidence of RID is also influenced by patient-related factors, such as high body mass index, nutritional status, smoking, alcohol, pre-existing skin disease, and genetic susceptibility (Böhner et al., 2020). As such, RID can be caused by a variety of factors and accounts for a high proportion of the adverse effects of conventional therapies in breast cancer patients, but there is currently no standard management or guideline for RID prevention (Wong et al., 2013; Chan et al., 2014). However, in clinical practice, topical managements, including moisturizing cream, are generally applied to patients with RID and their comparative studies have also been researched (Haruna et al., 2017; Ahn et al., 2020).

The water-in-oil type non-moisturizer cream, which consists of hyaluronic acid, shea butter, glycyrrhetinic acid, vitis vinifera, and telmesteine, was registered as a medical device for the symptomatic treatment of RID in the United States (US) and European Union (EU) (Primavera et al., 2006). Its components are believed to minimize radiation-induced skin reactions. All ingredients are powerful moisturizing agents that can attract and retain water under the skin barrier. In particular, hyaluronic acid, the main ingredient of the non-steroidal moisturizer, has demonstrated remarkable hygroscopic properties that are relevant to wound healing (Abramovits and Boguniewicz, 2006). Additionally, glycyrrhetinic acid has been reported to accumulate endogenous hydrocortisone and act as a natural steroid with anti-inflammatory properties (Kroes et al., 1997). Given the mechanism of these ingredients, the water-in-oil type non-moisturizer cream has shown significant efficacy at treating maximum severity skin toxicity, the severity of erythema, burning sensation within the radiation lesion, and desquamation, when compared to emollient base cream, which is similar in color and consistency to the moisturizer, but without the key ingredients (Leonardi et al., 2008). Therefore, it has been regarded as one of the available treatment regimens effective in the prevention of RID and the promotion of relief RT-related skin reactions.

Jaungo, a traditional herbal ointment consisting of *Lithospermi radix* and *Angelica sinensis*, is standardized based on the Korean Pharmacopeia. It has been authorized as a topical drug for dermatitis by the Korea Food and Drug Safety Administration (KFDA). In clinical practice, Jaungo has been used to treat a variety of dermatopathy symptoms, including RID. A few clinical trials have demonstrated the preventive effects of Jaungo on RID. A recent study found that Jaungo reduced the incidence of Gr ≥ 2 (46.7 versus 78.6%) and Gr 3 RID (20.0 versus 50.0%) without adverse events compared to general supportive skincare including gentle washing with neutral pH soap instead of prophylactic lotions or moisturizers, although it was not statistically significant (Kong et al., 2016). Additionally, brain tumor patients who underwent RT showed significant prevention of scalp dermatitis after topical application of Jaungo (Hayashi and Sato, 2011). In particular, shikonin, acetylshikonin, ferulic acid, and decusin, the major components of *Lithospermia radix* and *Angelica sinensis*, have been reported to have wound healing, antibacterial and anti-inflammatory effects (Huang et al., 2004; Hsiao et al., 2012; Ghaisas et al., 2014).

Given this background, we evaluated the comparative efficacy of Jaungo and the non-steroidal moisturizer in the prevention of RID in postoperative breast cancer patients. To our knowledge, this study is the first randomized controlled trial comparing topical agents to investigate the prophylactic efficacy between Jaungo ointment and the water-in-oil type non-steroidal moisturizer, which is one of the major clinical topical managements of RID for breast cancer patients with RID. This study can be considered as a noteworthy study protocol for highly prevalent RID without standard treatment, despite the limitations that double-blindness cannot be achieved, a pilot study with a small sample size, and the evaluation of outcomes may be subjective, depending on the assessors.

## Abbreviations

CSSP, catterall skin scoring profile; ECOG, eastern cooperative oncology group; HCG, human chorionic gonadotropin; NRS, numeric rating scale; RID, radiation-induced dermatitis; RTOG, radiation therapy oncology group; V, visit.
